# Biochemical typing of pathological prion protein in aging cattle with BSE

**DOI:** 10.1186/1743-422X-6-64

**Published:** 2009-05-26

**Authors:** Seraina Tester, Valerie Juillerat, Marcus G Doherr, Bianca Haase, Miroslaw Polak, Felix Ehrensperger, Tosso Leeb, Andreas Zurbriggen, Torsten Seuberlich

**Affiliations:** 1 NeuroCenter, Reference Laboratory for TSE in animals, Department of Clinical Research and Veterinary Public Health, Vetsuisse Faculty, University of Berne, Switzerland; 2 Institute of Genetics, Vetsuisse Faculty, University of Berne, Switzerland; 3 National Veterinary Research Institute, Pulawy, Poland; 4 Institute of Veterinary Pathology, Vetsuisse Faculty, University of Zürich, Switzerland

## Abstract

**Background:**

The broad enforcement of active surveillance for bovine spongiform encephalopathy (BSE) in 2000 led to the discovery of previously unnoticed, atypical BSE phenotypes in aged cattle that differed from classical BSE (C-type) in biochemical properties of the pathological prion protein. Depending on the molecular mass and the degree of glycosylation of its proteinase K resistant core fragment (PrP^res^), mainly determined in samples derived from the medulla oblongata, these atypical cases are currently classified into low (L)-type or high (H)-type BSE. In the present study we address the question to what extent such atypical BSE cases are part of the BSE epidemic in Switzerland.

**Results:**

To this end we analyzed the biochemical PrP^res ^type by Western blot in a total of 33 BSE cases in cattle with a minimum age of eight years, targeting up to ten different brain regions. Our work confirmed H-type BSE in a zebu but classified all other cases as C-type BSE; indicating a very low incidence of H- and L-type BSE in Switzerland. It was documented for the first time that the biochemical PrP^res ^type was consistent across different brain regions of aging animals with C-type and H-type BSE, i.e. independent of the neuroanatomical structure investigated.

**Conclusion:**

Taken together this study provides further characteristics of the BSE epidemic in Switzerland and generates new baseline data for the definition of C- and H-type BSE phenotypes, thereby underpinning the notion that they indeed represent distinct prion disease entities.

## Background

Bovine spongiform encephalopathy (BSE) is an infectious and fatal neurological disorder in *bovidae *and belongs to the group of transmissible spongiform encephalopathies (TSEs), so-called prion diseases [1]. Other examples of TSEs are scrapie in sheep and goats and Creutzfeldt-Jakob disease (CJD) in humans [2]. More than 20 years ago, BSE emerged in the British cattle population [3] and later, in most European countries [4], Japan [5] and North America [6]. Comprehensive epidemiological investigations identified contaminated meat and bone meal (MBM) that had commonly been used as an ingredient of concentrate feed as the vehicle that recycled the BSE agent in the cattle population [7]. However, the origin of BSE still remains under debate and it has been hypothesized that the disease derived from sheep scrapie, human TSEs [8] or from a spontaneous bovine prion disease analogous to sporadic forms of CJD in human [9]. Prion diseases are characterized by specific histopathological lesions and deposits of an abnormal conformational isoform (PrP^Sc^) of the host-encoded physiological prion protein (PrP^C^) in the CNS [10]. PrP^Sc ^but not PrP^C ^partially resists digestion by proteinase K (PK), resulting in an N-terminally truncated prion protein termed PrP^res^. The latter can be detected immunochemically, e.g. by Western blot (WB), in which it reveals a characteristic banding-pattern that reflects un-, mono- and diglycosylated PrP^res^- moieties. The apparent molecular masses and relative quantities of these glycoforms are used in biochemical PrP^res ^typing as the criteria to differentiate between prion diseases [11,12].

Until recently, BSE was thought to display uniform neuropathological [13] and biochemical features [14]. This changed in 2004, when BSE affected cattle identified in France and Italy revealed atypical higher (H-type) [15] or lower (L-type) molecular masses of PrP^res ^respectively in WB compared to classical (C-type) BSE. L-type BSE also differed from the C-type in the relative proportions of the PrP^res ^glycoforms and by PrP^Sc ^deposits in the form of amyloid plaques. It was therefore alternatively designated bovine amyloidotic spongiform encephalopathy (BASE) [16]. Both H- and L-type BSE were experimentally transmitted to mice [17] and cattle [18,19], resulting in phenotypes different from each other and from the C-type, thereby providing further evidence for the existence of at least three prion disease forms in cattle. L-type BSE has also been transmitted to mice transgenic for human PrP^C ^and these experiments pointed at distinctively higher transmissibility or even a higher zoonotic potential as compared to classical BSE [20,21]. In the meantime, some 40 H- and L-type BSE cases have been reported from various countries [22-24]. All of them were older, i.e. ≥ 8 years, compared to an average of 5–6 years in C-type BSE and were identified by means of active surveillance that targets *per se *clinically unsuspicious fallen and slaughtered cattle [25]. The sampling in active surveillance is usually restricted to the medulla oblongata as the primary target site for the diagnosis of C-type BSE and thus, with the exception of the two BASE index cases in Italy for which the complete brain was collected, biochemical characteristics of PrP^res ^in other brain regions of such atypical BSE cases could not be determined. Moreover, due to similar limitations this aspect has not yet been investigated in depth for C-type BSE, especially in aging cattle. It remains to be determined whether the biochemical features that currently serve for BSE phenotype definition are consistent when brain regions other than the medulla oblongata are investigated.

In Switzerland 463 BSE cases with an age range from 3.5 to 19 years have been diagnosed in cattle since 1990. In contrast to most other countries the majority of them were identified by passive surveillance, i.e. the reporting and laboratory confirmation of clinically suspicious animals. Most of these cases have not been subjected to any comparative biochemical analyses although in a considerable number suitable tissues from the medulla oblongata and other brain regions were collected.

In a recent study Jacobs and coworkers [23] proposed a systematic WB-based typing strategy to discriminate H-type, L-type and C-type BSE by using antibodies that specifically bind to the N- and C-terminal sequences and the core fragment of PrP^res^. Herein we adopt this strategy and analyze the PrP^res ^phenotype in up to ten different brain regions of 33 cases of BSE in aging indigenous cattle. Besides extending the baseline data on PrP^res ^phenotypes in BSE-affected cattle brains the results are expected to allow assessing the extent to which atypical cases are part of the BSE epidemic in Switzerland.

## Materials and methods

### Tissue samples

Suspected cases of clinical BSE (CS) were identified by passive surveillance, the animals were killed and their heads were forwarded to the NeuroCenter, Vetsuisse Faculty, University of Berne or the Institute of Veterinary Pathology, Vetsuisse Faculty, University of Zurich for statutory laboratory confirmation. In most cases, the complete brain was removed and split sagitally into two equal halves. One half was frozen at -20°C or -80°C and the other half was fixed in formalin. In the active surveillance program BSE cases were identified in emergency slaughtered (ES), routinely slaughtered (RS) or fallen cattle (fallen stock, FS) by approved BSE rapid tests in routine testing laboratories. Of these cases, with one exception (Elfe-06), only the medulla oblongata was available, divided similarly into two halves and forwarded to the NeuroCenter. All BSE cases included in this study were confirmed by histopathologic examination and/or immunohistochemical PrP^Sc ^detection in medulla oblongata sections as recommended by the World Animal Health Organization [26]. The L-type and the H-type BSE control material originated from Poland [27]. When available, the following brain regions of BSE positive cases were sampled from frozen tissue: medulla oblongata at the level of the obex (MO), cerebellar cortex (CC), midbrain (MB), hippocampus (HC), parietal lobe (PL), thalamus (TH), basal ganglia (BG), cortex frontalis (CF), cortex occipitalis (CO) and cortex temporalis (CT). Four different brain regions from clinically suspect but BSE unconfirmed animals (n = 39) were sampled whenever available: MO (n = 39), CC (n = 27), TH (n = 18), CF (n = 17). All tissue samples were homogenized at 10% (w/v) in homogenization buffer (Prionics) according to the manufacturer's instructions.

### Western immunoblot protocol I

WB analyses were based on a modified commercial BSE rapid test (Prionics Check Western, Prionics) and carried out essentially as described previously [23]. Briefly, 500 μl of homogenates (10% w/v) were digested with PK at 37°C for 1 h and PrP^res ^was precipitated by the addition of 650 μl of 100% isopropanol and subsequently centrifuged at 15'000 × g for 7 min. The resulting pellet was resuspended in 100 μl Lämmli-buffer (Bio-Rad), heated to 95°C for 5 min and stored at -20°C until use. Initially, 20 μl per sample were loaded on precast ten-well 12% NuPage Gels (Invitrogen). In case of strong positive signals, samples were further diluted in Lämmli buffer and reanalyzed until a clear banding pattern was observed. A biotinylated molecular mass marker (2 lanes, Sigma) and a C-type brainstem control sample from an average aged (5 years) C-type BSE affected cattle were included on each gel. After electrophoresis for 90 min at 150 V and transfer of the proteins to PVDF membranes (Millipore), the membranes were blocked with Prionics blocking buffer or 5% (w/v) non-fat dried milk in TBST. Samples were analyzed with three different PrP specific monoclonal antibodies (mAb): (i) core antibody 6H4 (_156_YEDRYYREN_164_, Prionics), 0.2 μg/ml TBST [28], (ii) N-terminal 12B2 (_101_WGQGG_105_), 0.2 μg/ml TBST [29] and (iii) C-terminal SAF84 (_175_RPVDQY_180_, CEA), 0.17 μg/ml TBST [30]. Polyclonal rabbit-anti-mouse-HRP (DAKO; 1:3'000 in TBST) was used as secondary antibody in combination with Streptavidin-HRP (Sigma; 1:20'000) that served to visualize the biotinylated molecular mass marker. Conjugate binding was detected by ECL plus (amershambiosciences) and exposure time to photographic films was from 15 sec to 4 minutes.

### PrP^res ^typing

The photographic films of the WB were digitalized on a flat-bed scanner, and the PrP^res ^signals were analyzed with the help of commercial software (Quantity One, Bio-Rad). Molecular masses and relative intensities of the un-, mono- and diglycosylated PrP moieties were assessed using mAb 6H4 in at least five independent WB runs. Average values and standard errors of the mean (S.E.M.) were calculated for each sample. To detect differences in the molecular mass of the unglycosylated PrP^res^, the average of all samples under investigation yielding a positive WB signal was calculated and cut-off limits were set at +/- 5%, as described previously [19]. With respect to the identification of putative L-type BSE, the cut-off for the relative intensity of the diglycosylated band was set to 55% [23]. The tri-plot excel template was downloaded from [31].

The reactivity of mAb 12B2 was compared with that of mAb 6H4 by analyzing all samples in duplicate in the same WB run, yet on separate gels and membranes. The first membrane was incubated with mAb 6H4, the second with mAb 12B2. Both were then exposed to the same photographic film and the signal intensities were assessed visually.

### Western immunoblot protocol II

An alternative WB format, the Bio-Rad TeSeE (WB protocol II) was performed as suggested by the manufacturer, but with Prionics Check Western homogenates as starting material, except for Charly-04 where 20% (w/v) homogenates in 320 mM sucrose were used. For 10% homogenates, the PK concentration was reduced to 50% in order to adjust the tissue/PK ratio to similar levels. MAbs Sha31 (_156_YEDRYYRE_163_, Bio-Rad, 1:10 – core antibody), 12B2 or SAF84 served as primary antibodies.

### PrioStrip and HerdChek BSE

The Prionics Check PrioStrip and the IDEXX HerdChek BSE tests are European Union- approved BSE screening tests and were used on 10% (w/v) homogenates according to the manufacturer's instructions.

### Deglycosylation

Deglycosylation was in principle performed as described by Biacabe and colleagues [7], and by using a commercial PNGase F kit (P07043, BioLabs). Briefly, the homogenates were digested as described in WB protocol II with the exception that the pellet was resuspended in denaturating buffer (4% sodium dodecyl sulfate, 2% β-mercaptoethanol, 192 mM glycine, 25 mM Tris and 5% sucrose). Subsequently, the denatured samples were treated with PNGase F as suggest by the manufacturer, mixed with 6× SDS PAGE sample buffer and analyzed by Western Blot according to protocol II.

### Genetic analysis

Identification of the 23 bp indel polymorphism (AJ298878.1:g.47836-47837ins23) and the 12 bp indel polymorphism (AJ298878.1:g49729_47730ins12) was carried out as described previously [32]. For analysis of the complete bovine PrP coding sequence, two overlapping fragments were amplified by PCR and directly sequenced on an ABI 3730 capillary sequencer (Applied Biosystems). Primer sequences and PCR- conditions are available upon request. The resulting sequences were assembled with Sequencer 4.8 (Gene Codes).

## Results

### BSE cases in aging cattle

Since H- and L-type BSE have been identified only in cattle ≥ 8 years of age, we extracted all such animals, in total 37, from our database that includes all confirmed BSE cases in Switzerland from the index case in 1990 until today. The surveillance stream, age, breed and available brain structures of these animals are compiled in table 1. In four cases (Karin-93, Linda-96, Nadia-04 and Ramona-05) suitable frozen CNS tissue had not been collected or was no longer available resulting in a total of 33 BSE cases to be included in the biochemical and genetic analysis. In active surveillance one animal was originally identified by immunohistochemistry, three animals by the TeSeE ELISA rapid test (Bio Rad) and the remaining by the Prionics Check Western test (data not shown).

**Table 1:** Cases of bovine spongiform encephalopathy in aged cattle (age ≥ 8 years) in Switzerland, as identified by passive surveillance (clinical suspect, CS) or active surveillance (emergency slaughtered cattle, ES; fallen stock, FS; regularly slaughtered cattle, RS) from 1990 to 2008 and the availability of frozen brain tissues samples from different neuroanatomical structures.

BSE case				Brain region sample availability^b)^									
	ID^a)^	Age	Breed^c)^	MO	CC	MB	HC	PL	TH	BG	CF	CO	CT

CS	Bambi-01	11.5	BV	X	X					X	X		
	Bärgi-97	8.4	SI/RH	X	X	X	X	(X)	(X)	X	(X)	(X)	X
	Bea-97	8.3	BV	X	X		X	X	X	X	X	X	X
	Charly-04	19	zebu	X	X		X	X^d)^	X^e)^	X	X	X	(X)
	Fortuna-00	8.1	HF	X	X		X	X	X	X	(X)	(X)	(X)
	Gabi-97	8.1	SI/RH		X		X	X			X	(X)	X
	Jalouse-99	8.9	SI/RH				X	(X)		X	X	X	X
	Julia-99	8.9	BV	X	(X)	X	(X)	(X)	X	X	X	(X)	X
	Karin-93	9	BV										
	Linda-96	10.2	BV										
	Loli-96	8	BV	X									
	Martina-96	8.9	SI/RH	X	(X)		X	X	X	(X)	X	(X)	(X)
	Meieli-99	9	SI/RH	X	X	X	X	X	X	X	X	(X)	X
	Mirelle-01	13.3	SI	X									
	Nadia-98	8.9	SI/RH		X	X	X	X	(X)	X	X	(X)	X
	Olga-98	8.1	SI/RH	X	(X)		X	X	X	X	(X)	(X)	X
	Orchidee-02	10.2	BV				X	(X)		X	(X)	(X)	(X)
	Priska-02	10.9	SI/RH	X									
	Spiegel-06	11.1	SI/RH	X	X	X	X	X	X	X	X	X	X
	Werita-98	8.3	HF		X	X	X	X	X	X	(X)	X	(X)
													
ES	Boheme-06	12.5	SI/RH	X									
	Dora-03	8.5	BV	X									
	Elfe-06	11	SI/RH	X	X	X	X	X	X	X	X	(X)	
	Elvira-01	11.9	SI/RH	X									
	Lilly-06	10.8	SI/RH	X									
	Nadia-04	11.4	SI/RH										
	Natascha-05	10.2	HF	X									
	Virginia-03	9	SI/RH	X									
													
FS	Berty-00	8.9	BV	X									
	Flurina-00	10.3	BV	X									
	Judith-02	8.8	BV	X									
	Ramona-05	8.7	BV										
	Starba-03	8.2	BV	X									
													
RS	Bunaug-02	14.6	BV	X									
	Carmen-01	9	SI/RH	X									
	Maya-03	9.4	HF	X									
	Ulla-04	8.7	SI/RH	X									

^a) ^animal name and year of diagnosis

^b) ^brackets indicate samples with signal intensities too weak for biochemical typing. MO, medulla oblongata; CC, cerebellar cortex; MB, midbrain; HC, hippocampus; PL, piriform lobe; TH, thalamus; BG, basal ganglia; CF, frontal cortex; CO, occipital cortex; CT, temporal cortex

^c) ^BV, Brown Swiss; SI/RH, Simmental/Red Holstein; HF, Holstein Friesian

^d) ^only available for TeSeE Western Blot

^e) ^only available for Check PrioStrip

### Biochemical typing confirms H-type BSE in a zebu

**Figure 1:**
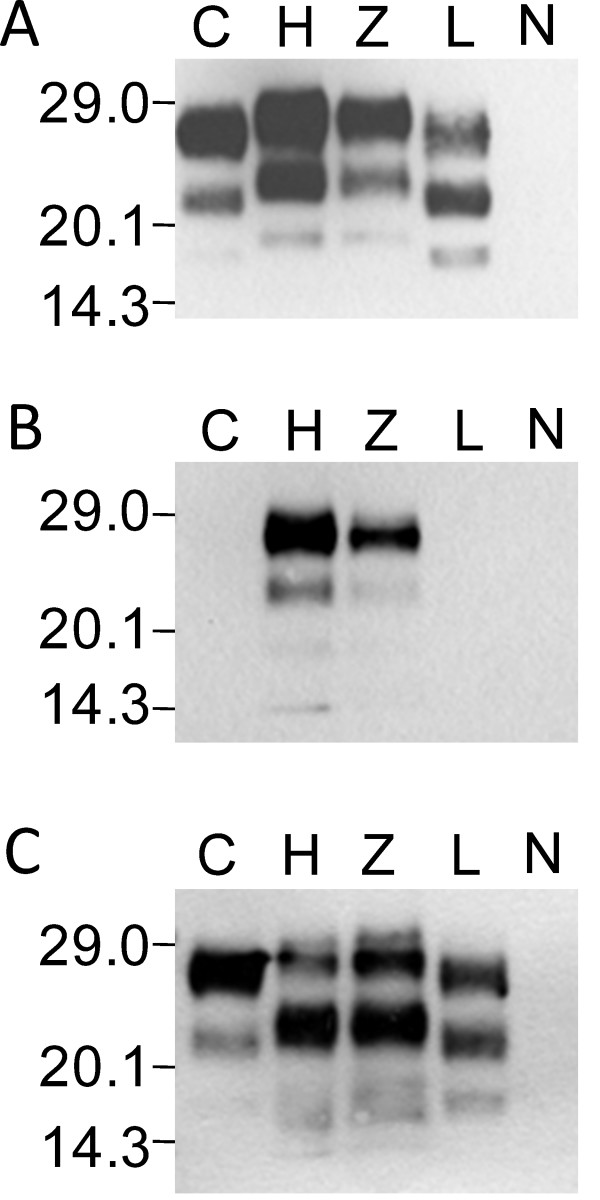
**Discrimination of BSE phenotypes by biochemical PrP^res ^typing**. Western blot profile of C-type BSE (C, 0.125 mg tissue equivalent per lane), H-type BSE (H, 2.5 mg), the zebu Charly-04 (Z, 3.33 mg), L-type BSE (L, 0.5 mg) and a BSE negative control (N, 2.5 mg) using a) a core-binding antibody (mAB 6H4) b) an amino-terminal-binding antibody (mAb 12B2) and c) a carboxy-terminal-binding antibody (mAb SAF84). Molecular masses of a marker are shown in kDa on the left.

In a previous study, we described a 19-years old spongiform encephalopathy affected cattle of the zebu breed (Charly-04) that presented biochemical PrP^res ^features distinct from classical BSE [33]. Here, we applied the PrP^res ^typing strategy (WB protocol I) first to a MO sample of this animal (table 1, Charly-04) in comparison to confirmed C- type BSE from Switzerland and H- and L-type BSE cases from Poland. Our results show, that (i) the molecular mass of the unglycosylated moiety of PrP^res ^was conspicuously higher in the zebu as compared to L- and C-type BSE and similar to the Polish H-type BSE control by using the PrP^res ^core-binding mAb 6H4 (Figure 1a), (ii) the N-terminal-specific mAb 12B2 readily detected PrP^res ^in the zebu and the H-type BSE control but not in C- and L-type BSE (Figure 1b), (iii) the C-terminal-specific mAb SAF84 revealed a complex banding pattern in the zebu and the H-type BSE control (Figure 1c) and (iv) the diglycosylated PrP^res ^moiety was predominant in C- and H-type BSE and the zebu using mAb 6H4, but not in the L-type control. Taken together these findings confirm that this methodology was appropriate to discriminate between the three BSE-types and that Charly-04 was indeed affected by H-type BSE.

### Atypical BSE in aging cattle in Switzerland

**Figure 2:**
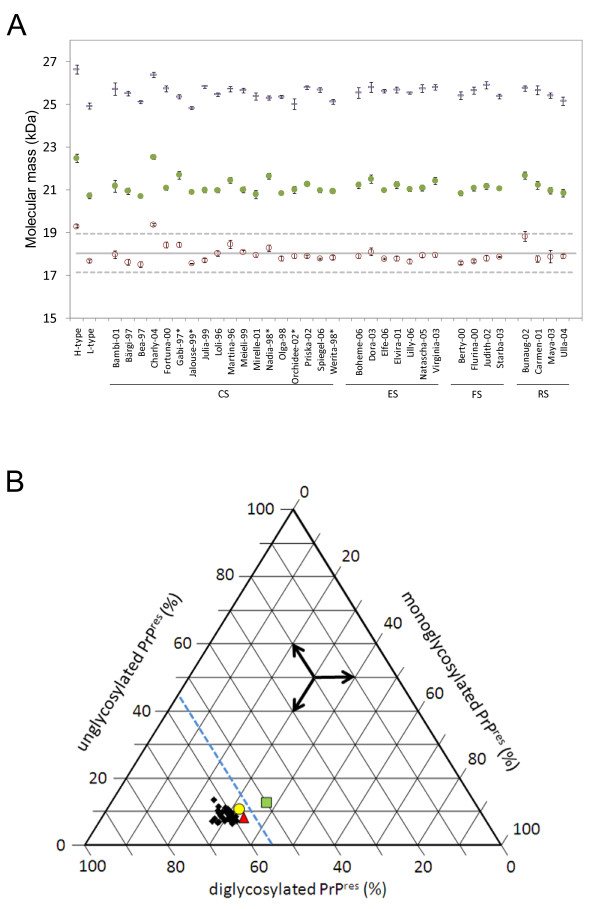
**Biochemical PrP^res ^typing in diagnostic target sites of aged cattle with BSE**. PrP^res ^was analyzed at least five times on different gels with a core-binding antibody (mAb 6H4) by Western blot. a) Average molecular masses of the unglycosylated (red open circles), the monoglycosylated (green filled circles) and the diglycosylated band (blue rectangles) of PrP^res ^are depicted with the related standard errors of the mean (S.E.M.). The cut-off value for the molecular mass of the unglycosylated band to discriminate H-type from C-type BSE was defined as the average molecular mass of all samples under investigation (solid line) +/- 5% (dashed line). b) Tri-plot graph presenting the relative intensities of the un-, mono- and diglycosylated PrP^res ^moieties. The L-type BSE control (green square) is the sole sample that fell below the decision limit of 55% relative intensity of the diglycosylated PrP^res ^(blue dashed line). The H-type BSE control is indicated by a red triangle and the zebu Charly-04 by a yellow circle. Samples were derived from medulla oblongata at the level of obex but where this was not available hippocampus was examined (marked by asterisks). CS, clinical suspect; ES, emergency slaughter; FS, fallen stock; RS, routine slaughter.

To investigate the PrP^res ^phenotype in detail we initially analyzed samples from the medulla oblongata (or from the hippocampus when MO was not available) in WB protocol I and we determined the molecular masses and relative proportions of the PrP^res ^glycoforms. Only the H-type control and the H-type zebu revealed an unglycosylated PrP^res ^band of an average molecular mass above 19 kDa with mAb 6H4. All other animals including the L-type control showed an average molecular mass in the range of 17.5 kDa to 18.82 kDa, which lies within the decision limit of ± 5% of the overall average molecular mass (Figure 2a). The average relative intensity of the diglycosylated band in all aging cattle with BSE was above the decision limit of 55% (57.5% to 64.9%) and for the L-type, as expected, much lower at 49.9% (Figure 2b). None of the cattle samples showed considerable reactivity with the N-terminal mAb 12B2 and all displayed a three-band pattern with mAb SAF84 (data not shown). Hence, the PrP^res ^phenotype of the remaining 32 Swiss cattle included in this study was uniform and in line with the characteristics of C-type BSE.

### PrP^res ^phenotype in C- and H-type BSE is conserved irrespective of the neuroanatomical structure

**Figure 3:**
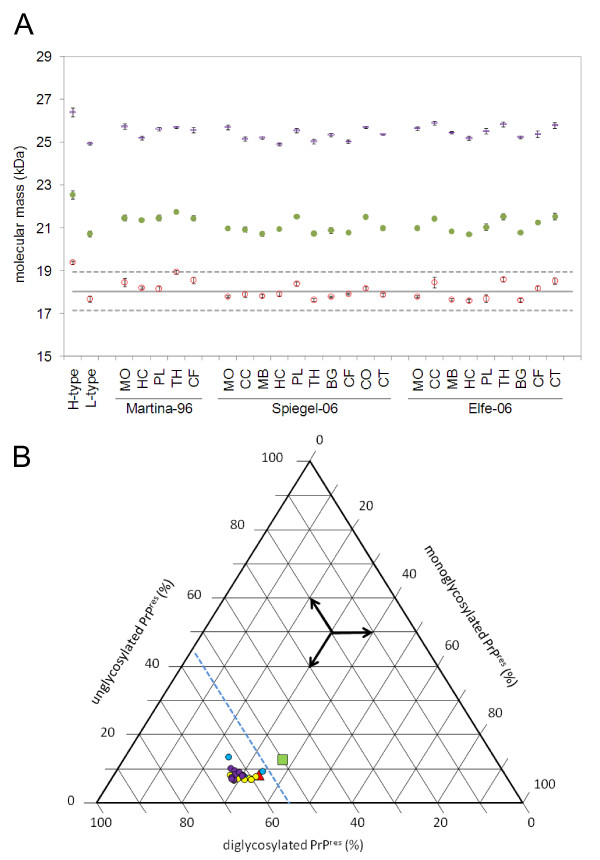
**Biochemical PrP^res ^typing in different brain regions of selected aged cattle with C-type BSE**. All analyses were conducted as described for figure 2. a) Molecular masses of unglycosylated (red open squares), monoglycosylated (green filled circles) and diglycosylated (blue rectangles) PrP^res ^and b) relative intensities of the un-, mono- and diglycosylated PrP^res ^moieties of the C-type BSE cases Spiegel-06 (yellow circles), Martina-96 (blue circles) and Elfe-06 (violet circles) as compared to L-type BSE (green square) and H-type BSE (red triangle). Whenever available, the following brain regions were analyzed: MO, medulla oblongata; CC, cerebellar cortex; MB, midbrain; HC, hippocampus; PL, piriform lobe; TH, thalamus; BG, basal ganglia; CF, frontal cortex; CO, occipital cortex; CT, temporal cortex.

**Figure 4:**
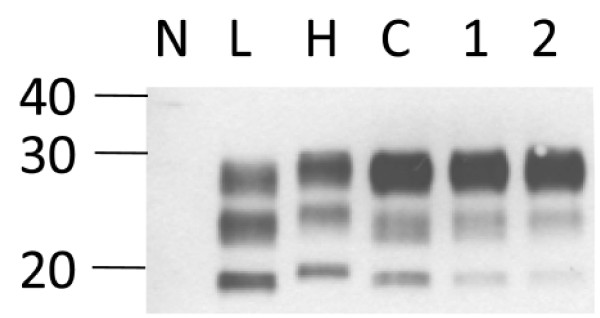
**Western immunoblot (protocol II) analysis of two ambiguous samples with mAb Sha31**. Samples from cases with BSE Martina-96 (lane 1, thalamus) and Bunaug-02 (lane 2, medulla oblongata) compared to L-type BSE (L), H-type BSE (Charly-04; H) and C-type BSE (C). Note that the unglycosylated PrP^res ^in both samples migrates in line with that in C-type BSE, and different from that in H- type BSE. On the left, a molecular mass marker is indicated in kDa.

**Figure 5:**
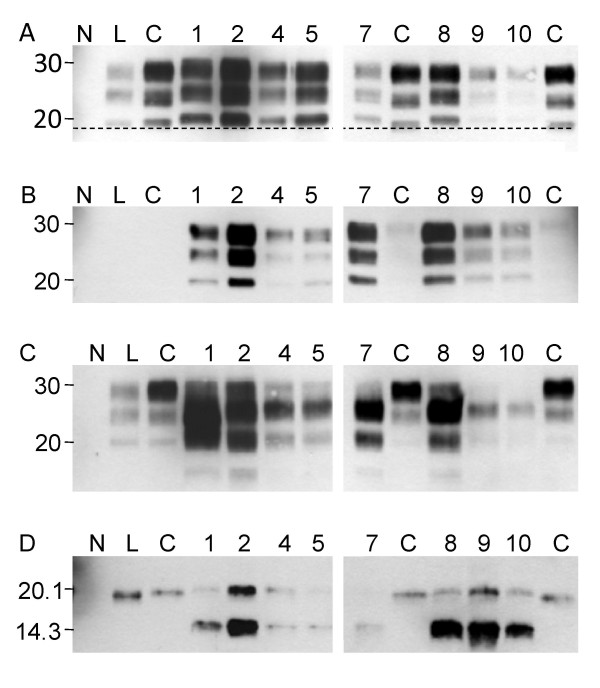
**Biochemical typing of different brain regions in H-type BSE**. Western blot analysis of the H-type BSE zebu (Charly-04) with a) a core-binding antibody (Sha31), b) an amino-terminal binding antibody (12B2) and c) a carboxy-terminal binding antibody (SAF84). Samples are assigned to the lanes as follows: negative control (N), L-type BSE (L), C-type BSE (C) and for the zebu medulla oblongata (lane 1, 15 mg tissue equivalent), cerebellar cortex (lane 2, 15 mg), hippocampus (lane 4, 0.75 mg), piriform lobe (lane 5, 15 mg), basal ganglia (lane 7, 1.5 mg), frontal cortex (lane 8, 15 mg), occipital cortex (lane 9, 15 mg) and temporal cortex (lane 10, 15 mg). The dashed line indicates the molecular mass of the unglycosylated C-type PrP^res ^and helps to visualize differences compared to the H-type BSE zebu. The same samples, but deglycosylated are shown in d) with a carboxy-terminal binding antibody (SAF84). A molecular mass marker (in kDa) is indicated on the left.

As we had access to brain tissues apart from the medulla oblongata in a series of the cattle with C-type and the zebu with H-type BSE (table 1), we analyzed for the consistency of the biochemical phenotype within these animals in up to ten different neuroanatomical structures per case. Regarding the molecular mass and the glycoform proportions (for examples see Figure 3 and for the complete data see Additional file 1) as well as the reactivity with mAbs 12B2 and SAF84 all cattle samples showed the characteristics of C-type BSE in all the brain structures (data not shown). Occasionally we observed a second band slightly above the unglycosylated one with mAb 6H4. However, this was not reproducible when we repeated the analysis starting from the same homogenate and resulted then in a pattern indicative for C-type BSE. In two others (Bunaug-02, MO, Figure 2; Martina-96, TH; Figure 3) the average molecular mass of the unglycosylated band was near to the upper decision limit. We decided to retest these samples in an alternative WB (WB protocol II) using mAb Sha31 as the core-specific antibody and a different PK digestion procedure. Here, both samples clearly revealed a molecular mass similar to C-type and not to H-type BSE (Figure 4). We also investigated the PrP^res ^phenotype in eight different brain regions of the H-type BSE affected zebu. Remarkably, all three criteria to identify H-type BSE, i.e. the higher molecular mass of unglycosylated PrP^res ^with the core antibody Sha31 (Figure 5a), the reactivity with mAb 12B2 (Figure 5b) and the complex banding pattern with mAb SAF84 (Figure 5c) were consistently fulfilled in the zebu irrespective of the neuroanatomical structure. After deglycosylation with PNGase F all brain regions investigated in the zebu revealed two bands at ~20.0 kDa and ~14.0 kDa while in C- and L-type BSE only one band at 18.5 kDa to 19.5 kDa could be identified with the C-terminally-binding mAb SAF84 (Figure 5d).

### Neuroanatomical PrP^res ^distribution in different types of BSE

**Figure 6:**
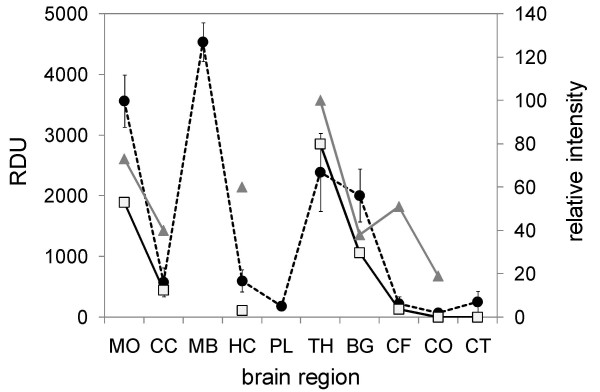
**Comparison of the PrP^res ^distribution in brains in different BSE phenotypes**. Tissue samples from all available brain regions in clinical suspect C-type BSE cases, the H-type BSE zebu and the emergency slaughtered C-type BSE case Elfe-06 were analyzed in a quantitative BSE screening test (Check PrioStrip). The average signal intensities (relative density units, RDU, left scale) in the C-type BSE cases (filled circles, dashed line) were compared to those of the H-type BSE zebu (open squares, continuous line) and to relative signal intensities (right scale) in Western blot analysis reported for experimentally infected L-type BSE cases (grey triangles) in the literature. For brain region code see figure 3.

PrP^res ^deposits in C-type BSE have been shown to be particularly intense in the MO at the level of the obex, the MB and the TH. To assess whether this is also true for aging cattle with C- and H-type BSE, PrP^res ^was measured in the tissue homogenates of the different brain regions in a commercial BSE rapid test (Prionics Check PrioStrip) that allows for a quantitative assessment of the PrP^res ^content. For comparison we also included quantitative Western blot data reported from experimentally infected L-type BSE in the literature [18]. Although for the H-type zebu the complete set of brain regions was not available, the overall PrP^res ^distribution corresponded well with that in the aging cattle with C-type BSE (Figure 6) and experimentally infected L-type BSE.

### Retrospective analysis of clinically suspicious but BSE unconfirmed cattle

Although some atypical BSE cases also showed histopathological lesions in the target sides that had been established for the diagnosis of C-type BSE [34] it is not known whether these structures are constantly affected, especially also in early stages of the disease. Moreover, for L-type BSE there is evidence that PrP^res ^accumulates not primarily in the obex region of the caudal brainstem, but rather in more rostral structures of the CNS [16]. Therefore, such cases may have been missed in the past, when the confirmation of clinically suspect cases relied mainly on the histopathological examination of the brain and later the detection of PrP^Sc ^by immunohistochemistry in the obex region. Samples from a total of 39 clinically BSE suspicious, but unconfirmed cattle with an age ≥ 8 years at the time of death derived from up to four different brain regions (MO, CC, TH and CF) per animal were investigated for the presence of PrP^Sc ^in a highly sensitive BSE screening test (IDEXX HerdChek BSE). None of the samples were positive, indicating that the initial diagnosis was correct.

### Genetics

We have shown previously that the susceptibility of cattle to BSE is associated to two polymorphisms, a 23 bp insertion/deletion (indel) and a 12 bp indel in the prion protein coding gene (*PRNP*) promoter region [18,35] and proposed a mechanism by which they may impact the expression rate of PrP^C ^in the host [36]. In the 33 BSE cattle under investigation, both polymorphisms were identified. To assess whether the allele-, genotype- and haplotype frequencies differed statistically from those in average aged BSE affected cattle (n = 76, age 5–6 years), we accomplished a cross tabulation with Chi-square (for 2 × 2 tables) and Fisher's exact test comparison. The results did not indicate any significant differences (p < 0.05) between these populations [see Additional file 2]. An H-type BSE case reported from the United States, a zebu crossbreed, had a mutation in the *PRNP *that had not previously been observed in cattle and encoded for lysine instead of glutamic acid at amino acid position 211 [37]. Sequencing of the *PRNP *open reading frame did not identify this or any novel polymorphisms in the Swiss aging BSE cattle including the H-type zebu.

## Discussion

Several studies aimed at describing the neuropathological phenotype of BSE cases in Switzerland in the past [38-41] and the pathological features in the cases investigated were found consistent with the principal phenotype of BSE reported from the United Kingdom [13,42] and elsewhere [43,44] without any evidence for unusual phenotypic features. The reports of atypical BSE cases in older cattle from many countries prompted us to target specifically aged BSE cases from Switzerland by a timely and validated biochemical typing strategy [23].

In the present study we characterize the biochemical PrP^res ^phenotype in 33 out of the total of 37 aging BSE cases that had been identified since the beginning of the epidemic in Switzerland in 1990. With the exception of one case that clearly fulfills the criteria of H-type BSE, all of them classify as C-type BSE. By analyzing in depth different brain structures in a large proportion of these cattle, we show for the first time that the PrP^res ^characteristics in both C-type and H-type BSE affected animals are conserved in structures outside the established diagnostic target site, the medulla oblongata.

Two cattle samples gave ambiguous, borderline results when the molecular mass of the unglycosylated PrP^res ^moiety was determined by WB protocol I with mAb 6H4. However, in an alternative approach (WB protocol II) that has also widely been used for PrP^res ^typing [24,45], no obvious differences compared to C-type BSE were found. Considering that these samples also revealed features of C-type BSE with C- and N- terminally binding antibodies we interpret these findings as electrophoresis artifacts in WB protocol I rather than a true difference in the biochemical phenotype. Contrary to some other reports, our results do not confirm a lower molecular mass of the unglycosylated PrP^res ^moiety in the L-type control sample as compared to C-type BSE [16,45]. However, some studies did not identify a significant difference either [19,23]. This discordance might be related to the digestion and electrophoresis conditions used in the respective laboratories and points to the importance to determine the relative intensities of the PrP^res ^bands to discriminate L-type from H- and C-type BSE.

In sporadic CJD remarkable biochemical heterogeneity has been described and frequently co-occurrence of distinct PrP^res ^types in different brain regions within the same patient was observed [46-49]. Whether the latter also applies to BSE has been poorly addressed in the past. Our results provide evidence that C-type BSE in aged cattle presents a much more stereotypic PrP^res ^phenotype, similar to what has been reported for variant CJD [46,47]. However, human CJD patients are in a much more advanced stage of disease at death compared to cattle and this situation may impact on the evolution of different types of PrP^Sc ^according to the brain region. By contrast, in H-type BSE the complex PrP^res ^pattern observed with C-terminal-binding antibodies has been shown to result from overlapping PrP^res^- triplet signals of two co-occurring types with apparent molecular masses of the unglycosylated moieties of ~20.0 kDa and ~14.0 kDa respectively [50]. This matches the findings in the H-type BSE affected zebu that consistently revealed both of these PrP^res ^signals and the other H-type specific features in all regional brain samples (Fig 5.). Also for L-type BSE (or BASE) WB analysis of two naturally [16] and two experimentally [18] L-type affected cattle showed that the biochemical phenotype was conserved between and within these animals irrespective of the brain region. Taken together, these and our data support the notion that the three bovine prion disease variants described to date appear as unique phenotypes and do not result from the variable co-existence of different prion strains within the same brain.

Genetic analyses identified no statistically significant differences between the frequencies of indel polymorphisms that have been associated with BSE susceptibility between the aged BSE cattle and an average-aged BSE control group. Thus, the relative late onset of disease in the aged cases might be related to other factors like the orally acquired infectious dose or the age at the time of infection.

In France [18,24] and Poland [27] the frequency of atypical BSE cases was remarkably constant in different birth cohorts and apparently not correlated to the number of cases in the C-type epidemic. These findings support the notion that H- and L-type BSE might represent sporadic prion diseases in cattle that occur spontaneously at a constant although low level in the population.

Exhaustive retrospective molecular typing studies of BSE cases in France [24] and Germany [19] estimated the prevalence of atypical cases as ~3.6 and ~3.0 cases per million tested cattle over 8 years of age respectively. From 1990 until 2007 ~130.000 aged cattle were targeted by passive and active BSE surveillance in Switzerland (table 2). Besides the H-type BSE affected zebu, all of the BSE cases included in the present study were of the C- phenotype. Indeed, under the assumption of a sporadic origin and prevalence equivalent to that in France and Germany, we would therefore expect less than one case of H-type and L-type BSE to be identified in the given sample-subset tested.

**Table 2:** BSE surveillance in Switzerland, 1990 to 2007.

Surveillance stream	Animals tested		Confirmed BSE cases	
	Total	Age ≥ 8y^a)^	Total	Age ≥ 8y
Passive surveillance	1,192	179	352	20
Active surveillance				
Emergency slaughter	70,237	10,536	39	8
Fallen stock	83,181	12,477	43	5
Regular slaughtered	718,857	107,829	29	4

Total	873,467	131,020	463	37

The number of totally tested and BSE positive confirmed animals and the fraction of cattle with a minimum age of eight years are shown and subdivided according to the surveillance stream.

^a) ^estimated numbers under the assumption that 15% of the tested animals had a minimum age of eight years (TVD-database, 2006; )

It must be emphasized that the Swiss BSE epidemic peaked in the mid 1990's, when cases were detected solely by passive surveillance. Its effectiveness depends largely on the level of disease awareness, a prerequisite to recognize diseased animals. While the clinical features of C-type BSE have been systematically documented [51], those for atypical BSE types remain unclear. The H-type zebu clearly displayed some signs indicative for BSE [33] and very recently experimental L-type [18,19] and H-type transmission proved to induce clinical, neurological disease in cattle. While C-type infected animals were nervous and hypersensitive, those challenged with L-type showed dullness accompanied by amyotrophic changes. As for C-type BSE we cannot rule out that a proportion of atypical cases were missed in the past, due to misinterpretation of clinical signs. But certainly, with the high level of disease awareness for BSE that we experienced in the past in Switzerland, we would expect that a large proportion of cattle that showed up with clear incurable CNS disease eventually resulted in BSE suspicion and confirmatory laboratory diagnosis.

The neuroanatomical PrP^Sc ^distribution and the absence of histopathological lesions in two fallen stock L-type cases in Italy [16,19] raised concerns whether histopathological examination and PrP^Sc^- immunohistochemistry applied to obex tissue sections are appropriate for laboratory confirmation of atypical BSE cases. The little data available suggests that at least at clinical stage H-type BSE ([33] and Figure 6), L-type BSE [18,52] and C-type BSE (Figure 6 and our unpublished data) involve prominent spongiform lesions and PrP^Sc^deposition in the obex and should therefore be readily identified also by conventional diagnostic techniques. To what extent this also applies to animals at a pre-clinical stage remains to be determined.

## Conclusion

Taken together these results indicate that the prevalence of H- and L-type BSE in Switzerland remains under the detection limit of the Swiss active surveillance program. However one H-type BSE case was identified by passive BSE surveillance and proves in principle the capacity to identify such cases in the population. Hence, the overall prevalence of atypical BSE in Switzerland appears very low and similar to what has been reported from other countries. It has been speculated and strengthened by experimental data [53,54] that atypical BSE once recycled in the cattle population was the origin of the worldwide BSE epidemic in the last 20 years. If this holds true and such cases occur spontaneously in the population, then BSE might never be completely eradicated. Furthermore, in these circumstances, it would be hazardous to relieve certain disease control measures, including the total prohibition of MBM in ruminant feed.

## Competing interests

The authors declare that they have no competing interests.

## Authors' contributions

ST and VJ conducted the biochemical typing experiments. BH and TL carried out the genetic studies. ST and TS drafted the paper. MP, FE, MGD and AZ contributed reference materials and tissue samples and/or critically revised the manuscript.

## Supplementary Material

Additional file 1 **Biochemical PrP^res ^typing in different brain regions of aging cattle with BSE**. The data provided present molecular masses and relative intensities of PrP^res ^moieties of all animals with more than one brain region available. Click here for file

Additional file 2 **Genetic analysis**. The data provided present allele, genotype and haplotype frequencies of the 23 bp indel and the 12 bp indel polymorphisms in geriatric compared to average aged BSE cattle. Click here for file
