# Supplementary material for: Biochemical typing of pathological prion protein in aging cattle with BSE
Source: Virol J. 2009 May 26;6:64. doi: 10.1186/1743-422X-6-64 (PMC2693104; doi:10.1186/1743-422X-6-64)
Supplement: Additional file 1 — Biochemical PrPres typing in different brain regions of aging cattle with BSE. The data provided present molecular masses and relative intensities of PrPres moieties of all animals with more than one brain region available. [file 1743-422X-6-64-S1.ppt]

## Slide 1
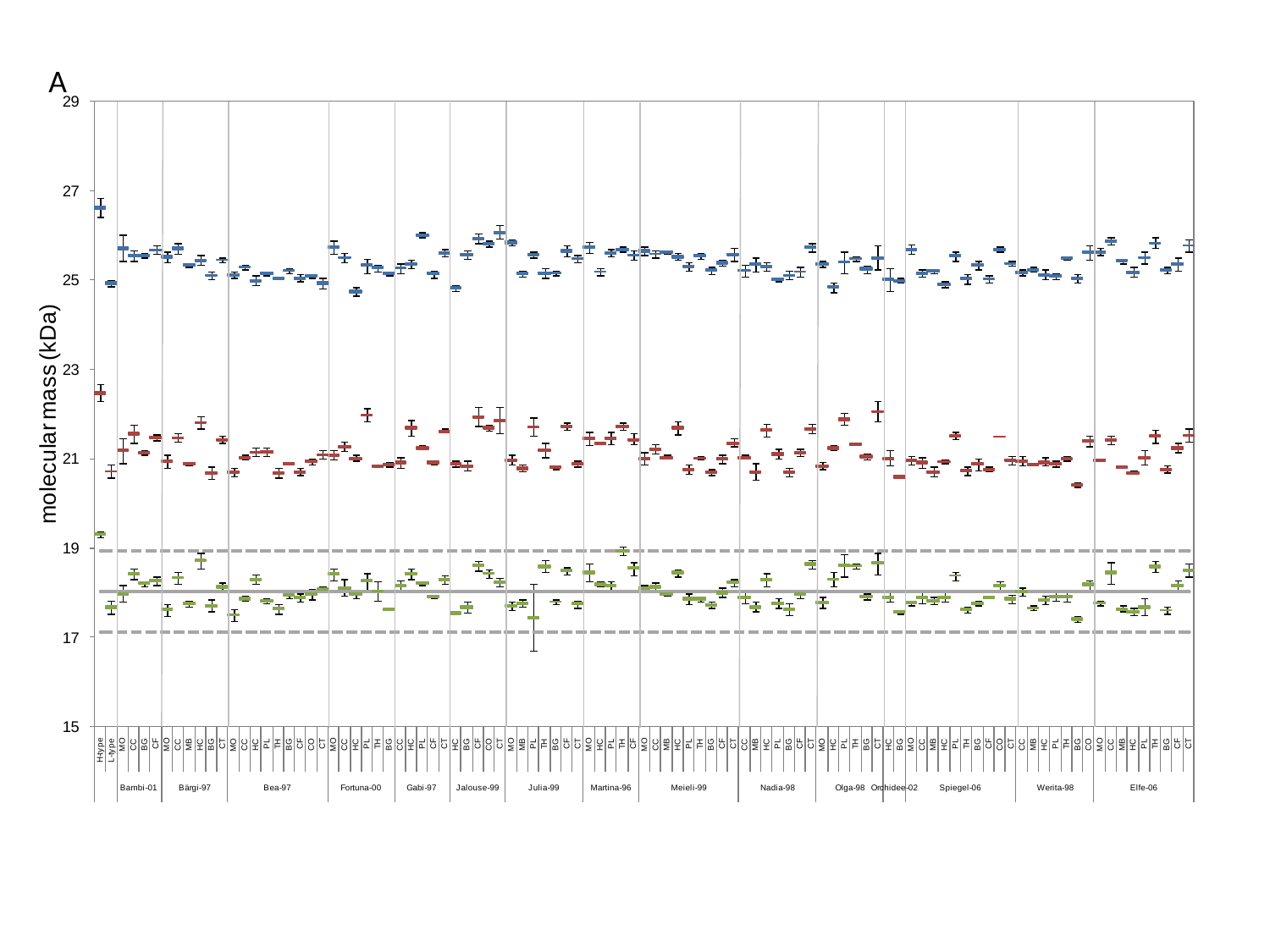

A

## Slide 2
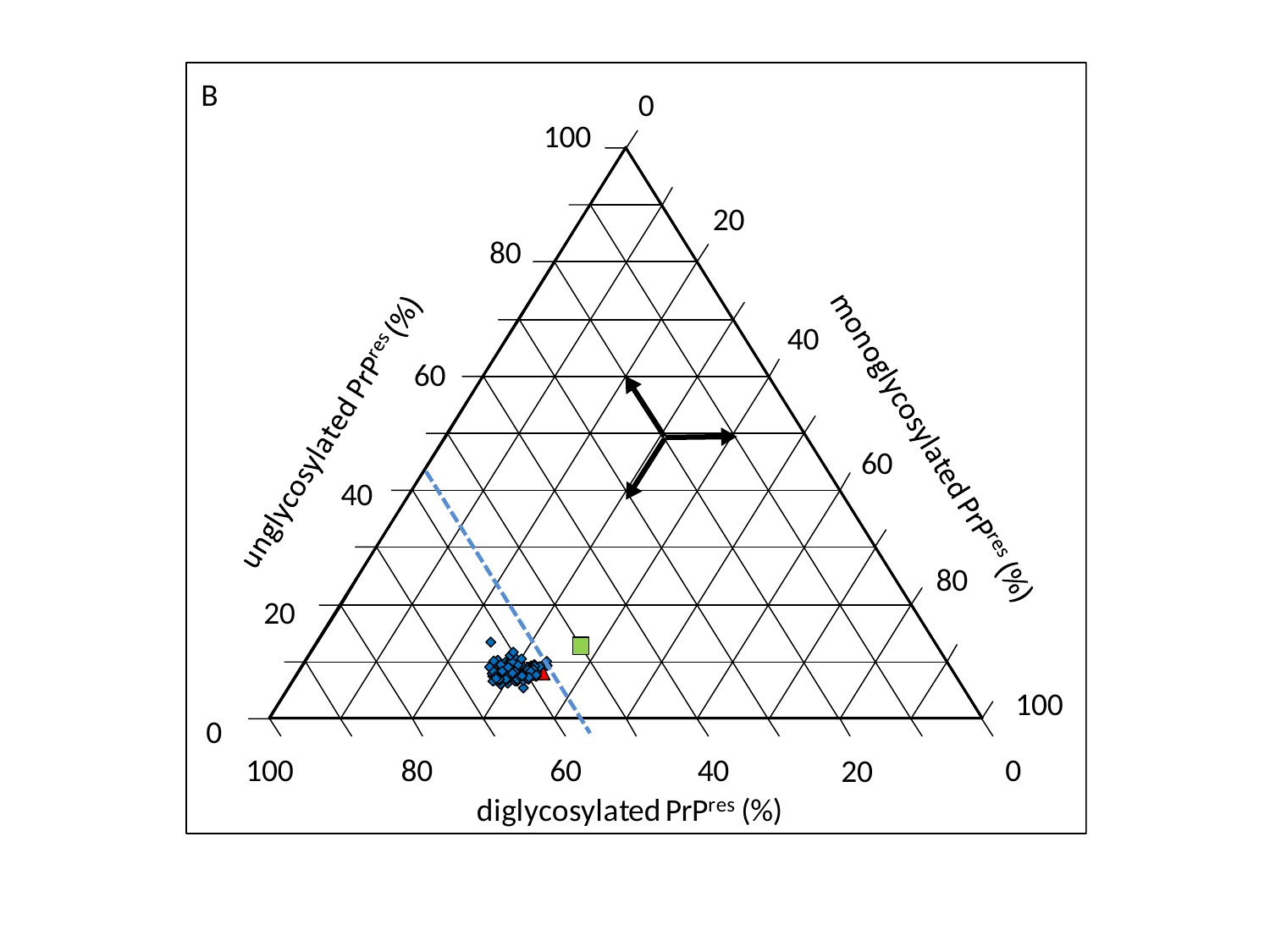

B

## Slide 3
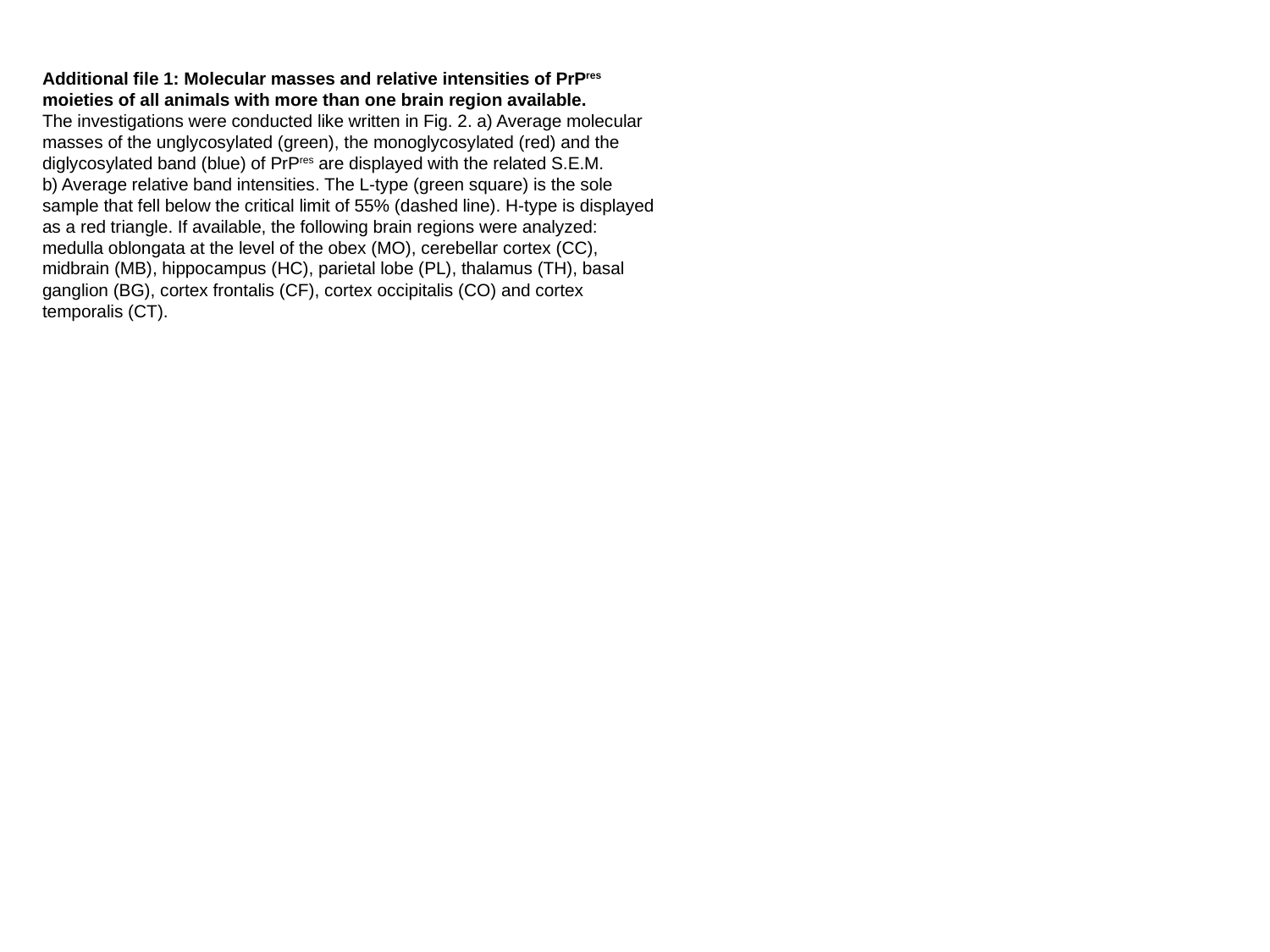

Additional file 1: Molecular masses and relative intensities of PrPres moieties of all animals with more than one brain region available.
The investigations were conducted like written in Fig. 2. a) Average molecular masses of the unglycosylated (green), the monoglycosylated (red) and the diglycosylated band (blue) of PrPres are displayed with the related S.E.M.
b) Average relative band intensities. The L-type (green square) is the sole sample that fell below the critical limit of 55% (dashed line). H-type is displayed as a red triangle. If available, the following brain regions were analyzed: medulla oblongata at the level of the obex (MO), cerebellar cortex (CC), midbrain (MB), hippocampus (HC), parietal lobe (PL), thalamus (TH), basal ganglion (BG), cortex frontalis (CF), cortex occipitalis (CO) and cortex temporalis (CT).
