# Supplementary material for: Biochemical typing of pathological prion protein in aging cattle with BSE
Source: Virol J. 2009 May 26;6:64. doi: 10.1186/1743-422X-6-64 (PMC2693104; doi:10.1186/1743-422X-6-64)
Supplement: Additional file 2 — Genetic analysis. The data provided present allele, genotype and haplotype frequencies of the 23 bp indel and the 12 bp indel polymorphisms in geriatric compared to average aged BSE cattle. [file 1743-422X-6-64-S2.ppt]

## Slide 1
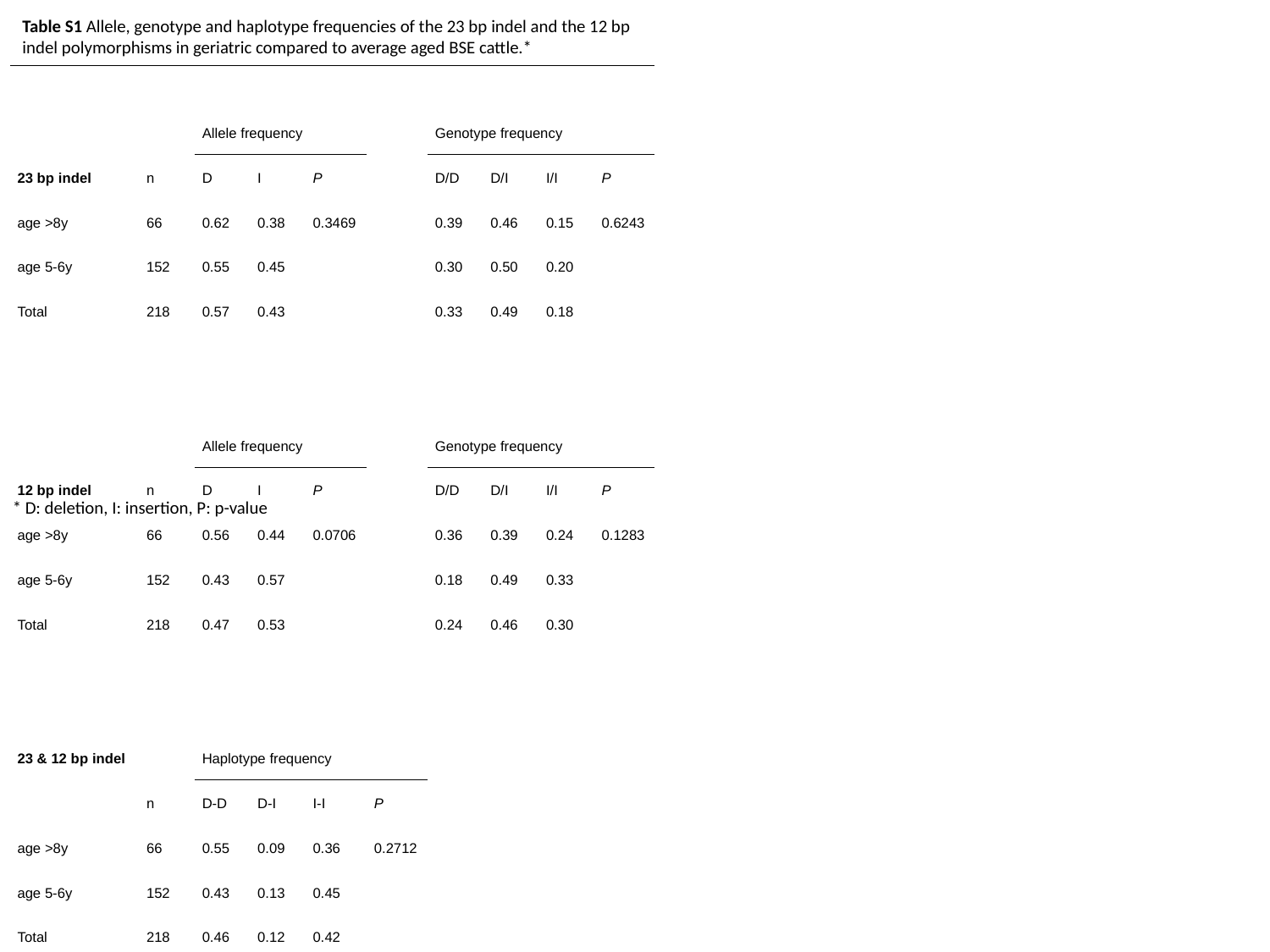

Table S1 Allele, genotype and haplotype frequencies of the 23 bp indel and the 12 bp indel polymorphisms in geriatric compared to average aged BSE cattle.*
| | | | | | | | | | |
| --- | --- | --- | --- | --- | --- | --- | --- | --- | --- |
| | | Allele frequency | | | | Genotype frequency | | | |
| 23 bp indel | n | D | I | P | | D/D | D/I | I/I | P |
| age >8y | 66 | 0.62 | 0.38 | 0.3469 | | 0.39 | 0.46 | 0.15 | 0.6243 |
| age 5-6y | 152 | 0.55 | 0.45 | | | 0.30 | 0.50 | 0.20 | |
| Total | 218 | 0.57 | 0.43 | | | 0.33 | 0.49 | 0.18 | |
| | | | | | | | | | |
| | | | | | | | | | |
| | | Allele frequency | | | | Genotype frequency | | | |
| 12 bp indel | n | D | I | P | | D/D | D/I | I/I | P |
| age >8y | 66 | 0.56 | 0.44 | 0.0706 | | 0.36 | 0.39 | 0.24 | 0.1283 |
| age 5-6y | 152 | 0.43 | 0.57 | | | 0.18 | 0.49 | 0.33 | |
| Total | 218 | 0.47 | 0.53 | | | 0.24 | 0.46 | 0.30 | |
| | | | | | | | | | |
| | | | | | | | | | |
| 23 & 12 bp indel | | Haplotype frequency | | | | | | | |
| | n | D-D | D-I | I-I | P | | | | |
| age >8y | 66 | 0.55 | 0.09 | 0.36 | 0.2712 | | | | |
| age 5-6y | 152 | 0.43 | 0.13 | 0.45 | | | | | |
| Total | 218 | 0.46 | 0.12 | 0.42 | | | | | |
* D: deletion, I: insertion, P: p-value
